# Surveillance of *Mycoplasma agassizii* in Texas tortoises (*Gopherus berlandieri*) for translocation with emphasis on treatment and recovery

**DOI:** 10.3389/fvets.2024.1525179

**Published:** 2025-01-17

**Authors:** Christin A. Moeller, Saren Perales, Wraith Rodriguez, Alynn M. Martin, Cord B. Eversole, Sandra Rideout-Hanzak, Paul Crump, Clayton D. Hilton, Scott E. Henke

**Affiliations:** ^1^Caesar Kleberg Wildlife Research Institute, Texas A&M University–Kingsville, Kingsville, TX, United States; ^2^Arthur Temple College of Forestry and Agriculture, Stephen F. Austin State University, Nacogdoches, TX, United States; ^3^Texas Parks and Wildlife Department, Austin, TX, United States

**Keywords:** danofloxacin, *Gopherus berlandieri*, lethargy, *Mycoplasma*, oxytetracycline, runny nose, stress, Texas tortoise

## Abstract

**Background:**

Texas tortoises (*Gopherus berlandieri*) are a Texas-state threatened species. Translocation is often suggested as a mitigation option; however, disease status and the potential for spread must be considered prior to such efforts. *Mycoplasma* infection of the upper respiratory tract is a concern within tortoise populations, which requires monitoring so translocation efforts do not inadvertently spread the disease.

**Objectives:**

We determined and compared the prevalences of *Mycoplasma agassizii* in Texas tortoises from donor and recipient sites in southern Texas prior to translocation, treated *Mycoplasma agassizii*-infected tortoises with danofloxacin, and developed alternate *Mycoplasma agassizii* treatments for Texas tortoises.

**Methods:**

We collected 171 and 23 Texas tortoises from a 270-ha and a 100-ha donor site and recipient site, respectively. We began a regimen of danofloxacin (6 mg/kg body weight injected subcutaneously every other day for 30 days) for tortoises with clinical signs (*N* = 20). We noted an additional 10 tortoises began displaying clinical signs of upper respiratory tract disease (URTD) after translocation, so we designed a trial to test tulathromycin (5 mg/kg body weight given intramuscularly once/week for 7 weeks) or oxytetracycline (8 mg/kg body weight given subcutaneously once/day for 14 days) as *Mycoplasma* treatments for symptomatic tortoises.

**Results:**

Within the donor and recipient sites, 56 (32.7%) and 8 (34.8%), respectively, had antibody titers suggestive of past exposure. Eighteen tortoises from the donor site (10.5%) and 2 from the recipient site (8.7%) displayed clinical signs (i.e., clear serous nasal discharge, conjunctivitis, and palpebral edema) consistent with Mycoplasmal URTD upon initial collection, even though all polymerase chain reaction (PCR) results were negative for active shedding of *Mycoplasma agassizii*. We ceased treatment after the first dose of danofloxacin due to adverse reactions, which only began to subside after 72 h from the initial dose. Neither tulathromycin or oxytetracycline caused the clinical signs of URTD to subside after a 50-day treatment period.

**Conclusion:**

*Mycoplasma* is a persistent issue facing Texas tortoises. Stressors, such as translocation, can cause *Mycoplasma*-seropositive tortoises to display clinical symptoms of URTD, which can abate without treatment, once the stressor subsides.

**Implications:**

Danofloxacin, the recommended treatment for *Mycoplasma* infection in tortoises, is too potent for Texas tortoises.

## 1 Introduction

The Texas tortoise (*Gopherus berlandieri*) is the smallest and most sexually dimorphic of six species of tortoises that are native to North America (1, 2). Within the United States, the Texas tortoise is found in southern Texas, in the region south from Del Rio to San Antonio to Victoria (2, 3), including the Lower Rio Grande Valley (LRGV). While their overall range is still intact, their abundance is thought to have declined and their distribution within the LRGV specifically has become sporadic and more restricted due to agricultural and urban development. Densities of Texas tortoises have been estimated to be as high as 35 tortoises/ha on lomas (4) [i.e., coastal wind-blown clay dunes (5)]. Studies conducted in grasslands and shrublands estimate densities as low as 0.26 tortoises/ha (6). Due to threats from illegal collection and commercial exploitation, Texas tortoises, were listed as a protected nongame species in Texas in 1977. Due to additional threats from habitat loss, particularly to high density loma habitats, the species is still in need of conservation action and applied management.

Texas tortoises can tolerate a broad range of habitat types, from lomas to grasslands and thorn scrub (4). They appear to reach their highest densities in loma habitat (7) and at lower densities, they can utilize relatively open-canopied or early successional habitats with increased light intensity at ground level and high herbaceous plant diversity (8). Texas tortoises are found in grasslands and shrublands of southern Texas and appear to tolerate grazing-induced brush encroachment (8).

However, development in the LRGV has been rapid in the last half-century. Conversion of large areas of native thorn scrub and coastal grasslands to agricultural, residential, and energy infrastructure land uses has occurred. For example, large-scale industrial development projects, such as SpaceX infrastructure and liquified natural gas (LNG) terminals, have resulted in the loss of habitat for Texas tortoises. To offset the loss of tortoise (*Gopherus*) habitat in other states, state wildlife agencies offer translocation as their mitigation strategy, even though results from translocations for gopher tortoises (*G. polyphemus*) and desert tortoises (*G. agassizii*) have varied (9–11). No formal assessment of the success of translocation in Texas tortoises has been conducted. One such obstacle before attempting translocation is determining the presence of pathogens and parasites within the donor and recipient populations. Without determining the health status of tortoises, it is possible to introduce a naïve donor population of tortoises to a disease-infected recipient population of tortoises, or vice versa. One such disease of concern in Texas tortoises is upper respiratory tract disease (URTD), which can be caused by the highly contagious, bacterial species *Mycoplasma agassizii* and *Mycoplasma testudineum* (12), with a possible third *Mycoplasma* bacteria identified by genomic sequencing from a desert tortoise (13).

*Mycoplasma* produces a variety of metabolites that cause dysfunction of the respiratory mucosal epithelial cells, and can migrate to the lungs and air sacs, leading to lung lesions that can result in pulmonary effusion (14). The bacteria cause a range of clinical signs including nasal discharge, swollen eyelids, lethargy, and a general failure to thrive, which can result in death. Tortoises have no diaphragm; thus, they cannot cough to expel a buildup of mucous in their lungs, which makes tortoises susceptible to respiratory infections (15). Chronic infections of *Mycoplasma* bacteria can cause lesions in the nasal cavities of tortoises (16), which can facilitate emaciation because tortoises locate food by olfaction, and lesions impair their ability to locate food (17). *Mycoplasma* infection was considered so detrimental to a threatened population of desert tortoises that Nevada Department of Wildlife instituted a euthanasia protocol for seropositive tortoises as a means to prevent transmission of the bacteria to naïve populations (18).

Danofloxacin is a third-generation animal-specific fluoroquinolone antibiotic that has been used to treat *Mycoplasma* infections in tortoises (19, 20). Compared with other antibacterial drugs, danofloxacin has stronger cell permeability, higher drug concentration in plasma and tissues, and stronger antibacterial activity that can exhibit an antibacterial effect even when the drug concentration is low (21). Danofloxacin is widely used in the treatment of respiratory diseases caused by *Mycoplasma*, *Actinobacillus,* and *Glaesserella parasuis* (22–24). Therefore, it is expected to have wide applications for controlling respiratory tract disease caused by *Mycoplasma* spp. (25).

Consequently, as part of a larger study investigating the efficacy of translocation for Texas tortoises, we examined the role of *Mycoplasma agassizii* in the translocation of Texas tortoises. Specifically, our objectives were to: (1) determine *Mycoplasma agassizii* prevalence in Texas tortoises from a donor site; (2) determine *Mycoplasma agassizii* prevalence in Texas tortoises at a recipient site; (3) compare prevalences between donor and recipient sites; (4) treat *Mycoplasma agassizii*-infected tortoises with danofloxacin; and (5) develop alternate *Mycoplasma agassizii* treatments for Texas tortoises.

## 2 Materials and methods

### 2.1 Study donor site

Our donor site was a 270-ha property located approximately 8 km west of Port Isabel, in Cameron County (26 1′38″ N, 97 14′53″ W), Texas, USA. The property is bordered to the north by Highway 48, which contains a 1-m tall impenetrable, cement barrier in the center of the highway, to the south by the Brownsville Ship Channel, to the west by a flooded drainage canal, and to the east by a perennial wetland. Hence, the donor site was essentially an island; thus, immigration and emigration was not possible. Eight habitat types, which included open water, wetland/riparian, coastal flats, shrubland, woodland, grassland, grassland loma, and evergreen loma, were identified on the property. Common plants found were Gulf cordgrass (*Spartina spartinae*), seacoast bluestem (*Schizachyrium littorale*), honey mesquite (*Neltuma glandulosa*), prickly pear cactus (*Opuntia* spp.), blackbrush (*Acacia rigidula*), coastal live oak (*Quercus virginiana*), common hackberry (*Celtis occidentalis*), Texan goatbush (*Castela erecta ssp. texana*), and non-native guineagrass (*Urochloa maxima*).

### 2.2 Study recipient site

Our recipient site was located about 200 km to the north-northwest of the donor site. The site was approximately 10 km south of Kingsville (27 28′21″N, 97 52′58″W), Kleberg County, Texas, USA. Both the donor and recipient sites were located in the Gulf Coast and Marshes ecoregion (26). Common plants found on the 110-ha recipient site included honey mesquite, huisache (*Vachellia farnesiana*), prickly pear cactus, granjeno (*Celtis ehrenbergiana*), guineagrass, and Kleberg bluestem (*Dichanthium annalatum*).

We built three, 2.4-ha enclosures to serve as isolation and soft release sites for translocated tortoises. We erected a 90-cm tall silt fence that had a 21 gauge, 2 × 2 cm wire back around the perimeter of each enclosure. The bottom 30-cm of the silt fence was buried in the soil and the plastic side faced inward to the enclosure so tortoises could not dig underneath or become entangled in the wire mesh. To prepare the recipient site to receive tortoises, we cleared brush and overgrown vegetation via mechanical removal (i.e., chainsaw and mowing), followed by prescribed fire, to develop a grassland with several mottes of honey mesquite trees scattered throughout each enclosure (8). Downed trees, areas of taller grasses around tree bases, and animal burrows were maintained as refugia for tortoises.

### 2.3 Duane M. Leach Research Aviary

The 700-m^2^ pavilion-style facility included 40, 1.2 × 1.8 × 2.0 m individual pens (Corners Unlimited^®^, Kalamazoo, Michigan 49001) made of 2.0 × 2.0 cm wire mesh, metal roof, and a concrete slab floor. The facility was open to the outside environment via the wire mesh walls, but sunlight was diminished due to the roof. Therefore, overhead lights were placed on timers to simulate daylength. Photoperiod of southern Texas fluctuates between 11 and 14 h of daylength with December and August having the shortest and longest days, respectively.^1^ The walls of each pen were lined with 22 mils vinyl-coated polyester tarp to eliminate direct contact between tortoises. Alfalfa hay was used as bedding and also could be eaten by tortoises. Tortoises were provided Mazuri^®^ tortoise low-starch, pelleted diet (Mazuri Exotic Animal Nutrition, St. Louis, Missouri 63166) and water *ad libitum*. We subsidized water intake by providing diced cucumber and watermelon every 2 days. Each pen was equipped with a 3-sided 40 (L) × 40 (W) × 20 (H) cm escape box with a wooden top for added shelter, a Fluker^®^ (Fluker Farms, Port Allen, Louisiana 70767) 150-W ceramic heat emitter bulb, and a Reptisun^®^ (Zoo Med Laboratories, Inc., San Luis Obispo, California 93401) UVA UVB Reptile 23 W fluorescent lamp. The heat lamps and UV lights were provided *ad libitum* and tortoises were free to move underneath or away from both devices as needed.

### 2.4 Tortoise collection and sampling

We conducted systematic searches, driving searches, detection dog searches, and incidental encounters for Texas tortoises from June – November 2022 at both the donor and recipient sites. Systematic searches consisted of 3–7 human searchers who walked from sunrise until 1,200 h and from 1,600 h until sunset, which coincided with the known activity pattern of Texas tortoises (4, 27). Driving searches used trucks and all-terrain vehicles (ATVs) along dirt roads and animal paths searching for tortoises. A detection dog (i.e., 12-year-old, female Labrador retriever) that was trained to locate Texas tortoises, was used to find tortoises at both sites. Lastly, incidental encounters occurred when we found a Texas tortoise while traveling to and from search locations within each site.

Tortoises were sexed, marked with an individualized number by filing grooves on the marginal scutes according to the methods of Cagle (28), weighed to the nearest gram, and carapace and plastron length, width, height, and circumference were measured (mm). Tortoise sex was determined based on external morphology such as length of the gular projection, plastral concavity, and the ratio of anal notch to anal fork width (29, 30). Tortoises whose sex could not be confirmed because of ambiguous characters were labeled as “unknown.” Due to difficulty in differentiating between male and female tortoises of small size based on shell morphology alone, tortoises <130 mm carapace length were categorized collectively as “juveniles” (31). Tortoise age was estimated from carapace length using the regression equations of Hellgren et al. (31) and Kazmaier et al. (6).

Tortoises located on the donor site were transported to the recipient site because the donor site was scheduled for immediate development. Tortoise health was visually examined according to methods of Berry and Christopher (32), especially noting clinical signs suggestive of URTD (e.g., nasal exudates, conjunctivitis, swollen eyes, labored/wheezy breathing), lesions suggestive of chronic URTD (e.g., nasal scarring and asymmetric nares), and lethargy (e.g., head and limbs limp, little-to-no resistance to having its head extracted from its shell, and lack of willingness to move when placed on ground); we recorded whether each of these clinical signs were either present or absent. Tortoises displaying signs of URTD were immediately transported to the Duane M. Leach Research Aviary for isolation.

Approximately 1.0 mL of blood was collected from either the caudal vein, brachial vein, or the subcarapacial venous sinus of each captured tortoise and placed in tubes containing lithium heparin (33). Blood samples were centrifuged, plasma collected, and frozen in vials at −80°C. Aseptic nasal irrigation was performed by injecting 0.5–1.0 mL of sterile saline in each naris, collecting the nasal discharge in a sterile container, and adding 0.5 mL of an enrichment medium (SP4 Glucose Broth, Remel, Lenexa, Kansas 66204), before freezing the solution at −80°C. Lastly, sterile rayon swabs (Puritan Medical Products Company LLC, Guilford, Maine, USA) were used to swab the caudal pharynx of each tortoise. Swab tips were separated and placed into sterile cryovials and frozen at −80°C. Frozen samples were shipped to the University of Florida for enzyme-linked immunosorbent assay (ELISA) testing, *Mycoplasma agassizii* culture, and polymerase chain reaction (PCR) testing as per the methods of Brown et al. (34) and Waites et al. (35). *Mycoplasma* species were determined based on a unique restriction fragment-length polymorphism fingerprint of the PCR amplicon (33, 34). ELISA titers of <1:32, 1:32, and ≥ 1:64 were considered negative, suspect, and positive, respectively (33). Culture and PCR results were classified as positive or negative for the presence of *Mycoplasma agassizii*.

### 2.5 Danofloxacin treatment

Tortoises (18 from the donor site and 2 from the recipient site for a total *N* = 20) that exhibited clinical signs consistent with URTD (described previously) were taken to the Duane M. Leach Research Aviary and placed in separate pens as previously described. Tortoises began a regimen of subcutaneous injections of danofloxacin (Advocin, Zoetis, Parsippany, NJ 07054) at 6 mg/kg every 48 h for 30 days, which is the documented treatment for chronic mycoplasmosis in *Gopherus* spp. tortoises (20). Tortoise daily response to treatment and food consumption was recorded.

### 2.6 Alternate *Mycoplasma* treatment design

We used 30 *Mycoplasma*-clinical Texas tortoises and 10 seemingly healthy tortoises in this study. We used the 20 original tortoises identified with URTD symptoms from the danofloxacin treatment and allowed those tortoises a 20-day acclimation period to recover from the danofloxacin.

During the acclimation period, we assessed tortoise health within our soft-release enclosures at the recipient site and found 10 additional translocated tortoises that were displaying clinical signs of URTD. The 10 additional tortoises displaying clinical signs of URTD did so within 45 days of translocation to the recipient site. In addition, we collected 10 non-symptomatic tortoises to use as controls.

The 30 symptomatic and 10 non-symptomatic tortoises were placed into separate and isolated pens within the Duane M. Leach Research Aviary. The non-symptomatic tortoises were placed in pens at the opposite end of the facility, and tortoise handlers used hand sanitizer and walked through a boot wash containing 2.6% sodium hypochlorite bleach solution to reduce the likelihood of contaminating healthy tortoises with *Mycoplasma*. The bleach of the boot wash was discarded and replaced daily.

We randomly divided the 30 symptomatic tortoises into 2 drug treatment groups and a control group, each containing 10 tortoises. Treatments were tulathromycin (Draxxin, Pfizer, Inc., New York, NY 10001) and oxytetracycline (Oxytet, Pfizer, Inc., New York, NY 10001). Tulathromycin was given intramuscularly once/week for 7 weeks at a dosage of 5 mg/kg body weight, and oxytetracycline was given subcutaneously once/day for 14 days at a dosage of 8 mg/kg body weight.

Stress can cause the onset of *Mycoplasma* symptoms, and handling of tortoises can be a stressor. Therefore, because of the different handling of tortoises due to the different drug prescriptions, we split each drug treatment group into two subgroups of 5 tortoises each. One subgroup of the tulathromycin group received the drug as recommended, and the other subgroup received the drug but also was handled as if in the other drug treatment group. For example, 5 tortoises received 1 tulathromycin injection once/week for 7 weeks; whereas, another 5 tortoises received the same tulathromycin injections in addition to being handled as if in the oxytetracycline group and received saline injections instead of oxytetracycline. In addition, two sets of control tortoises (i.e., 2 groups of 5 tortoises each) were used. One control set contained 10 symptomatic tortoises and the other set contained 10 non-symptomatic tortoises. Control tortoises were randomly assigned to handling schedule (Table 1). This design allowed us to compare the effects of 2 drugs with different handling schedules on a total of 40 tortoises (Table 1).

**Table 1:** Drug and handling schedule of 40 Texas tortoises (*Gopherus berlandieri*) to assess the effectiveness of tulathromycin (“Tula”) and oxytetracycline (“Oxy”) as treatments for *Mycoplasma*-induced upper respiratory tract disease.

	*Mycoplasma* study in Texas tortoises (5 tortoises in each group per treatment = 40 tortoises in total)							
	Group 1 (1X/wk × 7 weeks)		Group 2 (1X/day × 14 days)		Clinical sign (No trt)		No clinical signs (No trt)	
Day	Group 1A^1,4^	Group 1B^1,5^	Group 2A^2,4^	Group 2B^2,5^	Group 3A^3,4^	Group 3B^3,5^	Group 4A^3,4^	Group 4B^3,5^
0	Phlebotomy (Ab) and pharyngeal and nasal swabs (PCR) – all tortoises							
0	Tula	Tula	Oxy	Oxy	S	S	S	S
1		S	Oxy	Oxy		S		S
2		S	Oxy	Oxy		S		S
3		S	Oxy	Oxy		S		S
4		S	Oxy	Oxy		S		S
5		S	Oxy	Oxy		S		S
6		S	Oxy	Oxy		S		S
7	Tula	Tula	Oxy	Oxy	S	S	S	S
8		S	Oxy	Oxy		S		S
9		S	Oxy	Oxy		S		S
10		S	Oxy	Oxy		S		S
11		S	Oxy	Oxy		S		S
12		S	Oxy	Oxy		S		S
13		S	Oxy	Oxy		S		S
14	Tula	Tula		S	S	S	S	S
21	Tula	Tula		S	S	S	S	S
28	Tula	Tula		S	S	S	S	S
35	Tula	Tula		S	S	S	S	S
42	Tula	Tula		S	S	S	S	S
49	Phlebotomy (Ab) and glottal and nasal swabs (PCR) – all tortoises							

^1^Group 1 = Tulathromycin dosage, which was 5 mg/kg body weight given intramuscularly (IM) 1x/week for 7 weeks.

^2^Group 2 = Oxytetracycline dosage, which was 8 mg/kg body weight given subcutaneously (SC) 1x/day for 14 days.

^3Group 3 and 4 = No drug given.^

^4Groups A were handled as directed for the designated drug. Control tortoises in Group A were handled for the tulathromycin prescription.^

^5^Group B were provided the designated drug AND handled as if in both drug treatments but given saline injections (“S”) in place of other drug.

Serology and pharyngeal swabs were obtained as previously described within the *Tortoise collection and sampling* section prior to the start of the study and at the end of the 50-day trial. Frozen samples were shipped to the University of Florida for ELISA testing and PCR testing, as previously described. Tortoise general health and food consumption was recorded daily during the trial.

### 2.7 Data analysis

Chi-square analysis was used to compare frequencies of *Mycoplasma agassizii*-exposed tortoises between sexes (i.e., males and females) and sites (i.e., donor and recipient sites). Tests were considered significant at *p* ≤ 0.05.

## 3 Results

### 3.1 *Mycoplasma agassizii* prevalence at donor site

We collected 171 (72 M: 97F: 2 Juveniles) Texas tortoises from the 270-ha donor site (1 tortoise/1.6 ha density), of which 18 (10.5%; 8 M:10F) displayed symptoms of URTDs when first encountered, and 56 (32.7%; 26 M:30F) had titers suggestive of past exposure. Forty-one (73.2%), 14 (25.0%), and 1 (1.8%) Texas tortoises had titers that were 1:32, 1:64, and 1:128, respectively. The frequency of males and females with a history of *Mycoplasma agassizii* exposure did not differ (*χ*^2^ = 0.17, df = 1, *p* = 0.68). Adult tortoises, exclusive of the 2 juveniles (carapace length of 57 and 85 mm, respectively), had a mean carapace of 158.8 ± 7.4 mm (range = 134–208 mm) and were estimated to range from 6 to 19 years old (i.e., young adults). Of the 18 original tortoises that displayed clinical signs of URTD, 9, 5, and 4 displayed no, suspect, and low positive (1:64) titers, respectively, for *M. agassizi*. Of the additional 10 clinical tortoises 5, 3, 1, and 1 displayed no, suspect, 1:64, and 1:128 titers, respectively, for *M. agassizi*. All pharyngeal swabs and nasal irrigation samples were negative by culture and PCR methods to detect *Mycoplasma agassizii* bacteria.

### 3.2 *Mycoplasma agassizii* prevalence at recipient site

We collected 23 (16 M:7F) Texas tortoises from the 100-ha recipient site (1 tortoise/4.8 ha density), of which 2 (8.7%; 1 M:1F) displayed symptoms of URTDs when first encountered, and 8 (34.8%; 5 M:3F) had titers suggestive of past exposure. Five (62.5%), 2 (25.0%), and 1 (12.5%) Texas tortoises had titers that were 1:32, 1:64, and 1:128, respectively. The frequency of males and females with a history of *Mycoplasma agassizii* exposure did not differ (*χ*^2^ = 0.01, df = 1, *p* = 0.94). All tortoises collected from the recipient site were considered young adults (mean carapace length = 169.7 ± 5.9; range = 142–197 mm); estimated ages ranged from 8 to 18 years old. Of the two original tortoises that displayed clinical signs of URTD, 1 and 1 displayed no and low positive (1:64) titers, respectively, for *M. agassizi*. All throat swabs and nasal irrigation samples were negative by culture and PCR methods to isolate active *Mycoplasma agassizii* bacteria.

### 3.3 *Mycoplasma* comparison between donor and recipient sites

No difference in past *Mycoplasma agassizii* exposure (*χ*^2^ = 0.04, df = 1, *p* = 0.85) occurred between the donor (32.7% of 171 tortoises) and (34.8% of 23 tortoises) recipient sites. Both sites had tortoises that displayed low titers (≤1:128) of past exposure to *Mycoplasma agassizii*, with the majority of tortoises either negative (67 and 65% for the donor and recipient sites, respectively) or suspect (24 and 22% for the donor and recipient sites, respectively) of past exposure. Sex ratios (*χ*^2^ < 6.0, df = 3, *p* = 0.11) and age structures (*χ*^2^ < 0.2, df = 2, *p* = 0.89) were similar between the donor and recipient sites.

### 3.4 Danofloxacin treatment

Twenty Texas tortoises (18 from the donor site [10.5%] and 2 from the recipient site [8.7%]) displayed clinical signs (i.e., nasal discharge) consistent with URTD upon initial collection. These tortoises were placed into isolation at the Duane M. Leach Research Aviary where the signs of rhinorrhea continued. Within 3 h of the initial dose of danofloxacin, 7 of the 20 tortoises (35%) became listless, limbs and head became limp, eyelids were swollen and closed, and they had excessive salivation. After 20 h from the initial dose, 4 additional tortoises (i.e., an additional 20%) began displaying similar reactions to danofloxacin; however, the signs were not as severe as with the first seven tortoises. After 24 h following the initial dose, the reaction to danofloxacin by the 11 reactive tortoises had not subsided. Those tortoises were placed into a shallow tub (i.e., 40 × 40 × 2 cm) containing water to aid against dehydration due to the excessive salivation. The remaining 9 tortoises (45%) did not appear to have the severe reaction to danofloxacin; however, all tortoises stopped eating after the initial dose. By 48 h after the initial dose, reaction to danofloxacin had not diminished, so it was decided to terminate the danofloxacin treatment rather than continue with the second dose. By 72 h after the initial dose, excessive salivation began to clear and the tortoises’ eyelids were less swollen. Also, tortoise heads and limbs retracted back into their shells when stimulated. However, all treatment group tortoises appeared unable or unwilling to move. By the fifth day after the single dose of danofloxacin, tortoises began moving within their pens, and 16 of 20 began to eat their pelleted diet. Mortality caused by the single danofloxacin dose was not observed.

### 3.5 Alternate *Mycoplasma* treatment

At the beginning of the trial, morbidity was apparent within the 30 tortoises in the symptomatic groups because all tortoises were displaying rhinorrhea and raspy breathing, while the 10 tortoises within the non-symptomatic group continued to appear healthy. However, by the end of the drug trial, 22 tortoises were displaying signs of morbidity that were consistent with URTD, of which 8, 4, 6, and 4 tortoises were in the tulathromycin, oxytetracycline, symptomatic control, and non-symptomatic control groups, respectively (Table 2). Twelve of the previously symptomatic tortoises (40%) stopped displaying clinical signs during the drug trial; whereas, 4 (40%) of the non-symptomatic tortoises began displaying signs. One mortality occurred in a tortoise within the oxytetracycline treatment group that was additionally handled as a tulathromycin tortoise (Table 2).

**Table 2:** Number of Texas tortoises (*Gopherus berlandieri*), randomly assigned to a drug treatment group, that demonstrated various titers to past *Mycoplasma*-exposure before and after the drug treatment.

	Group A^1^			Group B^2^		
Titers^3^	Negative	Suspect	Positive	Negative	Suspect	Positive
Drug: Tulathromycin^4^						
Initial	2	3	0	5	0	0
Trial end	0	5	0	0	5	0
Drug: Oxytetracycline^5^						
Initial	0	3	2	3	2	0
Trial end	0	1	4^6^	3^7^	1	0
Clinical sign control:	Tulathromycin handling			Oxytetracycline handling		
Initial	2	3	0	2	3	0
Trial end	2	2	1	2	2	1
No clinical sign control						
Initial	4	1	0	4	1	0
Trial end	2	2	1	1	3	1

1 Group A were handled as directed for the designated drug. If a control tortoise, Group A were handled for the tulathromycin prescription.

2 Group B were provided the designated drug AND handled as if in both drug treatments but given saline injections in place of other drug.

3 Titers were considered Negative if < 1:32, Suspect if 1:32, and Positive if ≥ 1:64. Titers did not exceed 1:128.

4 Tulathromycin dosage was 5 mg/kg body weight given intramuscularly (IM) 1x/week for 7 weeks.

5 Oxytetracycline dosage was 8 mg/kg body weight given subcutaneously (SC) 1x/day for 14 days.

6 One positive titer reached the 1:128 dilution level.

7 One tortoise that was initially Negative died during oxytetracycline treatment.

By the end of the 50-day trial, titers increased (11/20, 55%) or remained stable (8/20, 40%) for each drug treatment, with the exception of one tortoise whose titer decreased (5%) from Suspect to Negative after treatment of oxytetracycline but experienced the handling of both drugs (Table 3). By contrast, 3, 2, and 5 of the symptomatic control tortoises improved (30%), remained stable (20%), and had increasing titers (50%), respectively, while 0, 4, and 6 of the non-symptomatic control tortoises improved (0%), remained stable (40%), and had increasing titers (60%), respectively, by the trial end (Table 3).

**Table 3:** Number of Texas tortoises (*Gopherus berlandieri*) that displayed rising titers to *Mycoplasma* (i.e., increased), or displayed stable or decreasing titers to *Mycoplasma* by the end of the 50-day trial.

	Tulathromycin^1^		Oxytetracycline^2^		Clinical sign (No trt)^3^		No clinical sign (No trt)^4^	
Serology^5^	Group 1A^6^	Group 1B^7^	Group 2A	Group 2B	Group 3A	Group 3B	Group 4A	Group 4B
Increased	2	5	3	1*	3	2	2	4
Stable	3	0	2	3	0	2	3	1
Decreased	0	0	0	1	2	1	0	0
Total	5	5	5	5	5	5	5	5

1 Tulathromycin dosage was 5 mg/kg given intramuscularly (IM) 1x/week for 7 weeks.

2 Oxytetracycline dosage was 8 mg/kg given subcutaneously (subQ) 1x/day for 14 days.

3 Clinical sign (No trt) were tortoises that displayed runny noses but were given saline injections to simulate treatments of tulathromycin (Group A) and oxytetracycline (Group B).

4 No clinical signs (No trt) were tortoises that appeared healthy but were given saline injections to simulate treatments of tulathromycin (Group A) and oxytetracycline (Group B).

5 Blood was acquired from the jugular vein of tortoises. An ELISA test was conducted to determine titers to Mycoplasma. Titers that remained stable or decreased from beginning to trial end were considered as potentially successful treatments.

6 Group A tortoises were handled as directed for their designated treatment AND as directed for the tulathromycin prescription.

7 Group B tortoises were handled as directed for their designated treatment AND as directed for the oxytetracycline prescription.

* Tortoise died on Day 38 of the 50-day drug trial.

No differences were observed (*χ*^2^ = 8.34, df = 6, *p* = 0.21) between the frequencies of tortoises with decreasing, stable, or increasing titers within the drug treatments and control groups. Nearly 50% of the *χ*^2^-value was due to a greater-than-expected number of tortoises within the symptomatic control group that experienced a decreasing titer from the initial to the end-of-trial serology. Prior to the drug trial, 22 (55%), 16 (40%), and 2 (5%) tortoises exhibited negative, suspect, or positive titers, respectively, which frequencies of tortoises with negative, suspect, and positive titers did not differ (*χ*^2^ = 11.6, df = 6, *p* = 0.07) between treatment groups. At the end of the trial, 9, 23, 6, and 1 tortoises exhibited negative and suspect titers. The frequencies of tortoises with negative, suspect, and positive titers did differ (*χ*^2^ = 13.9, df = 6, *p* = 0.03) between treatment groups. Nearly 50% of the *χ*^2^-value was due to fewer-than-expected within the tulathromycin group and more than expected within the oxytetracycline group having negative and positive titers, respectively.

Tortoise consumption of food varied by individual, but all tortoises ate pelleted feed during the trial and produced normal-appearing feces every 3 days, on average. Tortoises remained active during the trial, and no tortoise experienced excessive salivation after any injection of drug or saline.

*Mycoplasma agassizii* bacteria was not cultured from the throat swabs that were collected during the initial sampling or from the 50-day end-of-trial sampling. All *Mycoplasma agassizii* PCR results were negative.

## 4 Discussion

Clinical mycoplasmosis appears to have recently emerged as a problem in wild Texas tortoises in southern Texas. A wild population (*N* = 39) of Texas tortoises sampled from southern Texas during 2004 were all seronegative for *Mycoplasma agassizii* exposure; whereas 80% (12/15) of captive Texas tortoises housed at a rehabilitation facility in southern Texas were seropositive (36). Although seropositive tortoises were deemed non-releasable, the actual outcome history of the seropositive tortoises was not documented by Tristan (36). Guthrie et al. (37) documented that 11/40 (28%) and 3 additional (8%) Texas tortoises sampled from southern coastal Texas were antibody positive and suspect, respectively, for *Mycoplasma agassizii* exposure. Weitzman et al. (38) documented that 5 of 56 (9%) Texas tortoises from southern Texas were previously exposed to both *M. agassizii* and *M. testudineum*, 13 (23%) tortoises were only serapositive to *M. testudineum*, while the remaining tortoises (38 of 56, 68%) did not display previous exposure to *Mycoplasma* bacteria. Thus, mycoplasmosis may be a fairly new occurrence (i.e., within a decade) in Texas tortoises. Prevalence of past *Mycoplasma agassizii* exposure, titer levels, and percent of tortoises displaying clinical signs were consistent between our donor and recipient groups. Therefore, it does not appear that translocation of tortoises would cause the spread of *Mycoplasma agassizii* to a naïve population. However, it is prudent to assess prevalence and monitor current *Mycoplasma agassizii* outbreaks before translocation occurs.

*Mycoplasma testudineum* also has been documented to cause URTD symptoms. Due to financial constraints, we did not test for this species. Unfortunately, the most likely explanation is that the *Mycoplasma* symptomatic tortoises that tested negative for *M. agassizii* were infected by *M. testudineum*. Weitzman et al. (38) documented that Texas tortoises from southern Texas were exposed to both *M. agassizii* and *M. testudineum*; thus, there is precedent for such a situation.

We recognize that other causes, inclusive of viruses, colonic obstructions, foreign bodies, and trauma to the carapace, may have created the signs of URTD we observed. However, gastric reflux and foreign bodies were ruled out because tortoises did not display distended bowels, lacked green to brown saliva discharging from their mouths and nares, and were able to pass normal-appearing stools (39). Virus, such as herpes virus, ranavirus, adenovirus, reovirus, and paramyxovirus, can cause similar signs as *Mycoplasma* infection; however, tortoises often die quickly due to such viruses (40), which was not the case during our study. Coccidiosis caused by a protozoan parasite also can cause similar URTD signs in tortoises (39), but our tortoises displayed normal-appearing stools (i.e., no diarrhea); therefore, coccidiosis was ruled unlikely. Tortoises were examined upon capture and visual trauma to the carapace was not evident; thus, physical injury to the carapace was not involved in URTD signs displayed by tortoises during our study.

Even though we were unable to culture *Mycoplasma agassizii* bacteria via PCR, mycoplasmosis was still considered the most likely cause of our observed clinical signs. A negative PCR nasal flush does not necessarily mean that a tortoise is *Mycoplasma agassizii*-free (39). Both, *M. agassizii* and *M. testudineum*, grow slowly (2–8 weeks) at 30°C; thus, a tortoise may exhibit clinical signs of infection before high burdens of bacteria are present, limiting our ability to detect the presence of either pathogen using molecular methods, despite infection (12).

Although danofloxacin is offered as the current therapy to combat *Mycoplasma* infection in *Gopherus* spp., it does not appear to be appropriate for Texas tortoises. This may be because Texas tortoises are the smallest of the North American tortoise species, weighing half to one quarter, on average, of the gopher and desert tortoises, respectively (41). Although the dosage for danofloxacin is based on mg of drug per kg of body weight of tortoise, and the fact that Texas tortoises should have had a higher basal metabolic rate being a smaller tortoise, the severe reaction to a single dose of danofloxacin was concerning. Typical treatment would constitute 15 injections given every other day (19); however, tortoises required 3 days of recovery from the first dose for excessive salivation to cease. Because Texas tortoises are a threatened species (42), we feared excessive salivation for 30+ days would cause Texas tortoises to become dehydrated and die. Although we did not continue the danofloxacin treatment beyond the first dose; therefore, we cannot state with confidence that tortoises would have died if treatment continued. However, the apparent risk appeared too great for a threatened species.

Our attempt to develop a different treatment for *Mycoplasma* infection in Texas tortoises was unsuccessful. Oxytetracycline is a broad-spectrum tetracycline antibiotic used to treat infections caused by *Mycoplasma* organisms. It interferes with the ability of bacteria to produce essential proteins, without which, the bacteria cannot grow and multiply (43). Tulathromycin is a long-acting macrolide antibiotic that binds to the 50S ribosomal subunit within the RNA, which prevents bacteria from making vital proteins, and thus, keeps bacteria from multiplying (44). Tulathromycin has demonstrated efficacy against a diversity of respiratory pathogens in a variety of species, including reptiles (45).

A single mortality occurred during our alternate treatment method of oxytetracycline injections and additionally handled as within the tulathromycin group. This tortoise displayed typical URTD signs during treatment; however, did not appear to experience an extreme adverse reaction as did the tortoises given danofloxacin. Although only speculative, perhaps the extra handling (i.e., oxytetracycline injections plus additional saline injections of tulathromycin handling schedule) overtly stressed the tortoise, causing it to succumb to its URTD infection.

Our treatment regimens did not cease the clinical signs associated with URTD. However, we cannot fully state that treatment was ineffective because we were not able to culture sufficient bacteria to receive positive PCR results from our initial sampling. Initial positive PCR results followed by negative results after treatment would be indicative of an effective treatment. Interestingly, tortoises may not always shed bacteria, yet still display clinical signs, and may be subclinical yet be seropositive (45). Hence, we obtained potentially conflicting results (e.g., some tortoises displayed clinical signs and had rising titers for *Mycoplasma*, yet their PCR tests were negative). It is worth noting that tortoises within our study displayed very low titers (i.e., 1:32 [suspect] to 1:128), so cross-reactivity between the two species of *Mycoplasma* bacteria (i.e., *M. agassizi* and *M. testudineum*) could be possible to explain rising, but low, titers, yet samples be negative for PCR.

Some Texas tortoises displayed clinical signs of *Mycoplasma* infection after translocation, but the number of tortoises that did was lower than the number of tortoises found in the wild at the donor site with clinical signs of *Mycoplasma* infection. More studies are necessary to understand the reason for this. Possible explanations include the potential for a delayed response from additional translocated tortoises to develop symptoms, stressors at the site that would increase the frequency of symptomatic tortoises, or that translocation of tortoises is not any more likely to cause development of symptoms than the background symptom rate. Tortoises rarely clear *Mycoplasma* infections, and clinical signs can intensify and abate cyclically (45). Thus, the potential stressors of translocation and placement into an enclosure, placement into an isolated pen, and frequent handling by humans for treatment created situations that could have brought about clinical signs in some, but not all, tortoises. Although anthropomorphic, we believe our study demonstrates that Texas tortoises perceive and cope with various stressors differently. Hence, some seropositive tortoises remained subclinical throughout the study, while others displayed clinical signs throughout each aspect of the study.

In summary, *Mycoplasma* is an apparently common and persistent issue facing Texas tortoises. Stressors, such as translocation, can cause seropositive, but subclinical, tortoises to display clinical signs of URTD, but that rate was lower than the background rate observed in tortoises at the donor site. We believe it prudent to test for both *M. agassizii* and *M. testudineum* in Texas tortoises prior to translocation at both the donor and recipient sites. In addition, we believe it judicious to monitor the health of Texas tortoises after translocation to determine possible stress-related URTD effects. We caution against the use of antibiotics to combat mycoplasma infection in Texas tortoises, unless a strict monitoring plan is in place to offset potential side effects. Instead, we advocate that *Mycoplasma* clinical signs appear cyclic and can abate without treatment once the stressor subsides or the tortoise sufficiently copes with stress.

## Data Availability

The raw data supporting the conclusions of this article will be made available by the authors, without undue reservation.

## Footnotes

^1^ https://weatherspark.com/y/7960/Average-weather-in-Corpus-Christi-Texas-United-States-Year-Round
